# ‘They Even Have the Capacity, but the Places Aren't Prepared to Receive Them’: Community Environment and Participation of Autistic Children in a Family‐Engaged Research Analysis

**DOI:** 10.1002/aur.70316

**Published:** 2026-07-19

**Authors:** Léia Cordeiro de Oliveira, Cid André Fidelis de Paula Gomes, Gustavo Pietracatelli Janizello, Wallace Pereira Silva, Alice Soper, Monika Novak‐Pavlic, Olaf Kraus de Camargo, Soraia Micaela Silva

**Affiliations:** ^1^ Postgraduate Program in Rehabilitation Sciences Universidade Nove de Julho (UNINOVE) São Paulo SP Brazil; ^2^ CanChild—Centre for Childhood‐Onset Disability Research McMaster University Hamilton Canada

**Keywords:** autism, children, community, disability and health, international classification of functioning, participation, patient and public involvement, social environment

## Abstract

To examine the associations between community environmental barriers and support and the participation of autistic children, and to interpret these findings from the perspective of caregivers engaged as research partners. This cross‐sectional observational study employed a mixed‐methods design. The first phase comprised analytical and exploratory quantitative analyses. Caregivers of autistic children aged 5–12 years reported participation outcomes—frequency, involvement, and desire for change—using the Brazilian version of the Participation and Environment Measure for Children and Youth (PEM‐CY) and CARS‐BR with 80 caregivers of children aged 5–12. Phase 1 involved path analysis to test associations between community environment, income, autism severity, and participation (frequency, involvement, desire for change). Phase 2 employed a constructivist‐interpretivist approach, where five caregivers acted as research partners to interpret quantitative results via reflexive thematic analysis. Quantitative analysis showed no statistically significant associations between environmental factors and participation outcomes, despite frequent reports of barriers. In Phase 2, research partners identified social inclusion, safety, and logistical constraints as critical. They highlighted that low participation often stems from unwelcoming contexts and limited informal peer interaction, rather than a lack of desire to engage. Integrating findings from both phases, the study suggests that the low participation of autistic children in community settings may reflect, at least in part, protective choices adopted in response to contexts perceived as unwelcoming, unsafe, and insufficiently supportive, rather than a lack of desire for participation.

## 1 Background

In recent years, both the prevalence and the diagnosis of autism in children have increased substantially, leading to growing interest in research and in the development of diagnostic, assessment, and support strategies for this population (Zeidan et al. 2022; Singh 2021; Fernandes et al. 2023). The participation of children with neurodevelopmental conditions is influenced—positively or negatively—by multiple contextual characteristics, including environmental, physical, and social factors (Williams et al. 2019; Guichard and Grande 2019; Egilson et al. 2018).

According to Imms et al. (Imms et al. 2017), within the Family of Constructs Related to Child Participation, participation is a complex and multidimensional construct and is considered a central outcome in interventions, research, and educational practices. Participation may function both as a process and as an outcome and is essential to health and development across the lifespan. It is shaped intrinsically by factors such as competence, preferences, and sense of identity, and extrinsically by the context in which activities occur and by broader social and physical environments (Imms et al. 2017; Whybrow et al. 2025). This framework challenges linear models in which participation is viewed solely as a downstream consequence of changes in body functions or activity performance and highlights the role of environmental conditions in shaping participation opportunities and restrictions.

Research consistently indicates that autistic children experience distinct participation patterns compared with their neurotypical peers across home, school, and community settings, often characterized by differences in the frequency, diversity, and involvement in social and community‐based activities (Taheri et al. 2016; Egilson et al. 2017; Askari et al. 2015; Ratcliff et al. 2018). Ratcliff et al. (Ratcliff et al. 2018) reported lower participation in recreational and social activities among autistic children and adolescents and observed that these differences tended to become more pronounced with age. Similarly, Egilson et al. (Egilson et al. 2017) highlighted the importance of contextual factors in shaping participation experiences across everyday settings.

Previous caregiver‐reported studies have shown that participation among autistic children tends to be concentrated in home‐based activities and characterized by fewer opportunities for peer interaction and community engagement (Bedell et al. 2013; Lamash et al. 2020). These participation patterns are increasingly conceptualized as the result of dynamic interactions between the child and the environment, including contextual supports and barriers, consistent with the biopsychosocial perspective of functioning and participation. In community settings, environmental factors such as social attitudes, accessibility, and contextual demands have been identified as important influences on participation opportunities (Salters and Scharoun Benson 2026; Kaelin et al. 2024). In this context, instruments such as the Participation and Environment Measure for Children and Youth (PEM‐CY) have been widely used to simultaneously assess participation and environmental influences from the caregiver perspective, supporting more ecologically valid and family‐centered analyses.

These findings reinforce the need for context‐centered approaches to research and intervention. Although traditional assessments and interventions have predominantly focused on body functions and individual skills, they may not fully address priorities identified by autistic children and their families, which often emphasize meaningful participation in everyday activities and successful engagement across contexts (Coury et al. 2020; de Oliveira et al. 2025). Because participation occurs within family and community environments, the perspectives of autistic individuals and their families are essential for identifying relevant priorities and interpreting research findings (Eidson et al. 2020; den Houting et al. 2022; Verdecias‐Pellum et al. 2025; Kwon et al. 2018; Retamal‐Walter et al. 2023). Patient and family involvement in research has gained international recognition for promoting more relevant, ethical, and community‐responsive science through shared decision‐making and collaboration throughout the research process (Jesus et al. 2025; Novak‐Pavlic et al. 2023). Engaging families as collaborators rather than solely as research participants provides opportunities to address priorities that directly affect everyday life and to promote change informed by lived experience (Jesus et al. 2025; Novak‐Pavlic et al. 2023; Frisch et al. 2020).

Because community environments encompass family contexts, research and intervention goals targeting autistic children should be developed in partnership with autistic individuals and their families (Eidson et al. 2020; den Houting et al. 2022). Evidence suggests that autistic individuals and their families value such involvement, express interest in participating, and are capable of contributing meaningfully to research and decision‐making processes (Eidson et al. 2020; den Houting et al. 2022; Verdecias‐Pellum et al. 2025; Kwon et al. 2018; Retamal‐Walter et al. 2023). The involvement of patients and families in research has gained international recognition for its contribution to more relevant, ethical, and community‐responsive science, emphasizing shared decision‐making (Jesus et al. 2025; Novak‐Pavlic et al. 2023). Nevertheless, in some contexts, the social needs and preferences of children and families remain insufficiently addressed in research and clinical practice. Engaging families as collaborators rather than solely as research participants offers an opportunity to address issues that directly affect them and to promote change informed by lived experience (Jesus et al. 2025; Novak‐Pavlic et al. 2023).

Collaborative research involving family members, patients, healthcare professionals, and researchers supports the integration of multiple perspectives, the establishment of meaningful priorities, and critical dialog across all stages of the research process, from the formulation of research questions to the translation of findings into practice, with the ultimate goal of improving health and well‐being (Jesus et al. 2025; Novak‐Pavlic et al. 2023; Frisch et al. 2020). Accordingly, the aim of this study was to examine how community environmental barriers and supports are associated with the participation of autistic children and to interpret these findings from the perspectives of family members engaged as research partners.

## 2 Methods

### 2.1 Study Design

This cross‐sectional observational study employed a mixed‐methods design, comprising analytical and exploratory quantitative components and descriptive qualitative components, grounded in a constructivist–interpretivist paradigm.

In the first phase, parents or primary caregivers of autistic children aged 5–12 years completed the PEM‐CY through individual interviews, during which a trained researcher read each item aloud and recorded the participants' verbally reported responses. This phase adhered to the Strengthening the Reporting of Observational Studies in Epidemiology (STROBE) guidelines (Malta et al. 2010), ensuring methodological rigor and transparency throughout the observational process.

In the second phase, an interpretive meeting was conducted with caregivers of autistic children aged 5–12 years who had not participated in the initial data collection. These caregivers were engaged as research partners and constituted an advisory board, collaborating in the interpretation of the study findings. Caregivers who had not participated in Phase 1 were intentionally invited to ensure a more reflexive and independent interpretive process, minimizing potential bias associated with prior exposure to the data collection procedures and instrument. Drawing on their lived experiences, they contributed contextual insights that enriched the understanding of the results. Their reflections generated subthemes that were incorporated into the results. A follow‐up meeting was held after study completion to inform partner families about the discussions, conclusions, and the integration of their contributions into the final study outcomes (Jesus et al. 2025; Novak‐Pavlic et al. 2023).

The study protocol was approved by the Research Ethics Committee of Nove de Julho University (CAAE: 70424923.1.0000.5511). Written informed consent was obtained from all participating parents or legal guardians prior to data collection. Research partners involved in the interpretation phase signed a confidentiality agreement as research collaborators, as unpublished study data—including graphs displaying means and percentages—were shared and discussed during the interpretive meeting.

### 2.2 Participant Recruitment and Eligibility

Participants were parents or primary caregivers of autistic children aged 5–12 years with a formal diagnosis according to DSM‐5 criteria established by the American Psychiatric Association (American Psychiatric Association 2013), encompassing all three levels of support needs. Individuals were excluded if they declined participation, did not complete the full assessment protocol, or if the child had severe comorbid neurological conditions or genetic syndromes (e.g., cerebral palsy, epilepsy, or known genetic syndromes) that could substantially impact functioning beyond autism. These criteria did not exclude common co‐occurring conditions associated with autism (e.g., ADHD or anxiety). Information regarding diagnoses was obtained through parental report during screening, based on prior medical evaluations.

Recruitment followed a convenience sampling approach at the two facilities where the study was conducted. Eligible caregivers were approached in person while accompanying their children during scheduled therapy sessions. Before recruitment, all potential participants received general information about the study objectives through printed materials and verbal explanations provided by staff members. At the time of recruitment, trained researchers provided additional details, addressed questions, and obtained written informed consent from those who agreed to participate. Caregivers not involved in Phase 1 were purposively recruited to promote an independent and reflexive interpretation of the findings, minimizing potential bias associated with prior exposure to the study procedures and instrument.

### 2.3 Study Size

Sample size estimation was based on correlation results between environmental factors and participation outcomes obtained from a pilot study involving the first 10 participants. Calculations were conducted assuming a significance level of *α* = 0.05, statistical power of 80% (*β* = 0.20), and an expected correlation coefficient of *r* = 0.50. The estimated sample size was 51.68 participants. To account for a potential 30% attrition rate, the final target sample size was set at a minimum of 68 participants (Miot 2011).

### 2.4 First Phase: Data Collection

Demographic data were collected using a structured assessment form developed collaboratively by the study authors. Information obtained for parents or caregivers included age, sex, marital status, educational level, and family income. Data collected for children and adolescents included sex, age, duration since diagnosis, and school attendance.

Autism severity was classified using the Childhood Autism Rating Scale—Brazilian Version (CARS‐BR) (Pereira et al. 2008), categorizing children as having mild/moderate or severe autism. Participation and environmental factors in the community context were assessed from the caregivers' perspective using the PEM‐CY (Coster et al. 2011), translated and culturally adapted for Brazilian Portuguese (Galvão et al. 2018). Authorization to use the instrument was obtained from CanChild. Assessments were administered by two physiotherapists with at least 5 years of professional experience and specialization in pediatric practice, who received standardized training in the application of the instrument.

### 2.5 PEM‐CY—Brazilian Version

The PEM‐CY is a caregiver‐reported instrument developed by CanChild (Coster et al. 2011) and culturally adapted for use in Brazil (Miot 2011). Grounded in the International Classification of Functioning, Disability and Health (ICF), the PEM‐CY assesses children's participation and environmental factors across three contexts: home, school, and community. The instrument focuses on actual participation rather than independence in task performance, capturing engagement regardless of the use of assistive devices, support, or environmental adaptations.

For the community setting, the participation section comprises 10 activity items. For each activity, caregivers report:

- Frequency of participation, rated on an 8‐point scale (0 = *never* to 7 = *daily*);
- Level of involvement, rated on a 5‐point scale (1 = *minimally involved* to 5 = *very involved*);
- Desire for change, indicated as yes/no, followed by the extent of desired change, rated from 0% to 100%.

The environmental section comprises 16 items for the community setting, focusing on physical, cognitive, and social demands. For each item, caregivers indicate whether the environmental factor represents a support (“usually helps”), is neutral (“it's not a problem”), or acts as a barrier (“usually makes harder”). Environmental supports and barriers were operationalized as separate summary measures rather than summed ordinal scores. Specifically, the number of items classified as supports and the number classified as barriers were each divided by the total number of items answered and multiplied by 100, yielding two percentage scores: Environmental Supports (%) and Environmental Barriers (%).

### 2.6 Variables and Covariates

Variables were organized into dependent (endogenous) and independent (exogenous) categories, based on a conceptual model informed by the ICF and family‐centered frameworks.

#### 2.6.1 Dependent Variables

Participation outcomes were assessed using PEM‐CY scores and treated as continuous variables. The following participation dimensions were analyzed as dependent variables: (i) frequency of participation, reflecting how often the child engages in age‐appropriate activities; (ii) level of involvement, capturing the degree of engagement, motivation, and emotional connection during participation; (iii) desire for change, representing caregiver‐reported wishes for changes in the child's participation patterns.

#### 2.6.2 Independent Variables

The following predictors were included as independent variables: Family income, categorized into three brackets according to Brazilian socioeconomic parameters: (i) Income 1: up to R$ 2900; Income 2: R$ 2901 to R$ 7100; Income 3: R$ 7101 to R$ 22,000. (ii) Environmental factors derived from the PEM‐CY were operationalized as two separate percentage scores: Environmental Supports (%) and Environmental Barriers (%), calculated as the proportion of items classified by caregivers as supports or barriers relative to the total number of items answered.

#### 2.6.3 Covariates: Personal and Diagnostic Characteristics

To control for potential confounding effects on participation outcomes, the following personal and clinical characteristics were included as covariates: (i) Child's age (years; continuous variable), to account for developmental differences in participation; (ii) Child's sex (male/female), included as a dichotomous variable; (iii) Parental education level (years of formal schooling), used as a proxy for socioeconomic and cultural resources influencing participation opportunities; (iv) Autism severity, assessed using the CARS‐BR.

### 2.7 Second Phase—Family Engagement Approach

This study was guided by the principles of Family Involvement in Research (Cross et al. 2024), which emphasizes collaboration between researchers and families as equal partners across different stages of the research process. The Engagement Matrix (Smits et al. 2020) was used to define the roles of research partners, according to which they actively contributed not only during the execution of the study but also to the analysis and discussion of the findings, offering insights grounded in their lived experiences.

During the interpretive phase, five family members with no prior research experience, who had not participated in the data collection, contributed to contextualizing and refining the interpretation of the quantitative results obtained from the PEM‐CY. Their involvement included familiarization with the study objectives and design, presentation of the quantitative findings in graphical formats, and guided discussions to explore questions, uncertainties, and meanings arising from the results.

The engagement process was structured to promote mutual respect, shared learning, and co‐interpretation of findings. A semi‐structured discussion guide was used to organize key topics derived from the quantitative results, facilitate open dialog, and enable caregivers to interpret and contextualize findings related to participation, environmental supports and barriers, and desire for change, drawing on their lived experiences. The meeting was conducted by a single trained researcher, and the guide served as a flexible framework, with follow‐up probes used to elicit deeper reflections and additional insights when needed. Individual meetings with the research partners lasted between 40 and 60 min. The interviews were conducted virtually via Google meet, and all sessions were transcribed verbatim for analysis.

To support this process, the first author and the corresponding author received prior training in family engagement. The first author completed the international Family Engagement in Research (FER) program developed by the CanChild—Centre for Childhood‐onset Disability Research while the corresponding author completed its first Brazilian offering delivered at the Federal University of Juiz de Fora (UFJF). This training included core principles of family engagement, family‐centered research approaches, and strategies to support collaborative and participatory research practices.

This collaborative approach is aligned with the Canadian Institutes of Health Research (CIHR) Strategy for Patient‐Oriented Research (SPOR) and the CanChild Family Engagement in Research Framework, reinforcing the ethical and scientific relevance of involving families in the interpretation of research findings.

The family partner meeting guide included the following questions:

– When you look at these results, do they resonate with your daily experiences?
– Is there anything in these findings that surprises you or does not seem to reflect families' realities?
– What do you think might explain the observed results (e.g., do the identified facilitators and barriers reflect your daily experiences and your desire for change)?
– Which environmental factors do you believe most strongly influence the participation of autistic children in daily activities?
– Do you think anything was overlooked in the questions posed to caregivers or in the way the interviews were conducted?

### 2.8 Data Analysis—First Phase

Data distribution was assessed using the Shapiro–Wilk normality test. Sample characteristics were described using descriptive statistics, with means and standard deviations for normally distributed continuous variables and frequencies for categorical variables. For variables with nonnormal distributions, medians and interquartile ranges were reported. Radar charts were used to illustrate caregiver‐reported participation (frequency and level of involvement) and desire for change, while environmental supports and barriers were presented using frequency graphs.

Before conducting the main quantitative analyses, preliminary linear regression models were fitted to examine the individual associations between each covariate (child age, sex, parental education level, and autism severity) and participation outcomes in the community setting. Only covariates that showed statistically significant associations (*p* < 0.05) with at least one participation domain were retained for inclusion in the path analysis models. This strategy was adopted to reduce model complexity and minimize the risk of overfitting, ensuring that the final models focused on the most relevant predictors of participation.

Path analysis was subsequently performed to examine the direct associations between environmental factors and participation outcomes, in accordance with a theoretically grounded and statistically parsimonious model informed by the ICF framework. This approach enabled the simultaneous evaluation of multiple predictors. For each outcome treated as an endogenous variable, two path analysis models were specified, including all variables with potential exogenous effects. Analyses were conducted using the PATHj module in Jamovi, an interface for the *lavaan* package (version 2.6.23) (Rosseel 2012).

Model adequacy was assessed using the likelihood ratio test and information criteria, allowing comparison between path analysis and regression models. Models were estimated using the maximum likelihood (ML) method. Model fit was evaluated using Bentler's comparative fit index (CFI), Steiger–Lind's root mean square error of approximation (RMSEA) as an index of parsimony, and the standardized root mean square residual (SRMR) as an indicator of predictive accuracy. The coefficient of determination (*R* ^2^) was used to estimate the proportion of explained variance in the endogenous variables, with 95% confidence intervals reported where applicable.

### 2.9 Data Analysis—Second Phase

In the second phase, statements provided by the five family research partners were analyzed using a reflective and inductive thematic analysis, following the approach proposed by Braun and Clarke (Braun and Clarke 2019). One researcher conducted the initial coding of all transcripts, identifying and coding meaningful excerpts. This process was followed by iterative discussions with two additional researchers, who contributed to refining the coding structure and supporting the development of themes and subthemes. During this stage, some subthemes were revised, merged, or renamed to enhance conceptual coherence. Once the final set of codes, themes, and subthemes was established, their relationships were examined and discussed collaboratively by the full research team (Pyo et al. 2023; Im et al. 2023).

## 3 Results

A total of 84 caregivers of autistic children initially consented to participate in the study. Of these, two were excluded due to incomplete interviews, and two were excluded because the child had a comorbid diagnosis of Down syndrome. The final sample comprised 80 caregivers.

The mean age of participating caregivers was 39.69 years, and the majority were female (91.3%). The autistic children had a mean age of 6.89 years, with males representing 80% of the sample. Regarding clinical characteristics, 51.2% of the children had received their autism diagnosis between 3 and 5 years before the study, and 66.3% were classified as having mild to moderate autism severity according to the CARS‐BR. Additional demographic and clinical characteristics are presented in Table 1.

**TABLE 1:** Demographic data of children and young individuals with autism.

Variable	*N*/average	%/±
Child gender		
Male	64	80.0
Female	16	20.0
Child age (years)	6.89	±2.18
Child diagnostic time (years)		
0–1	7	8.8
1.1–2	23	28.7
3–5	41	51.2
6–10	9	11.3
CARS		
Mild/moderate autism	53	66.3%
Severe autism	27	33.8%
Child school		
No	7	8.8
Particular	26	32.5
Public	47	58.7
Respondent relationship to child		
Mother	61	76.3
Father	10	12.5
Others	9	11.2
Respondent age (years)	39.69	±7.42
Respondent work		
Yes	43	53.8
No	37	46.2
Family monthly income		
Up to 2.9 thousand	23	28.7
R$ 2.9–7.1 thousand	38	47.5
R$ 7.1–22 thousand	17	21.3
Higher than 22 thousand	2	2.5
Respondent marital status		
Single	27	33.8
Married	43	53.7
Divorced	8	10.0
Widowed	2	2.5

*Note:* Income brackets expressed in Brazilian Reais (R$). Data presented as mean ± standard deviation or absolute frequency and percentage.

Abbreviation: CARS: Childhood Autism Rating Scale—Brazilian Version.

Preliminary linear regression analyses were conducted to examine the associations between personal and clinical factors and each participation domain. No statistically significant associations were identified between child age, sex, parental education level, or autism severity as measured by the CARS‐BR and the participation outcomes of frequency and involvement (all *p* > 0.05), as presented in Table 2.

**TABLE 2:** Results of linear regression models for each participation domain (*n* = 80).

Participation domain	*R* ^2^	Predictor	*β* coefficient	*p*
Involvement	0.038	Age	0.0103	0.769
		Sex	0.0188	0.922
		Parental education	−0.0013	0.983
		CARS	−0.2694	0.101
Frequency	0.072	Age	−0.0961	0.303
		Sex	0.0810	0.874
		Parental education	−0.2407	0.133
		CARS	−0.4928	0.256
Desire for change	0.040	Age	0.9041	0.779
		Sex	−1.3224	0.940
		Parental education	−9.6572	0.083
		CARS	3.2551	0.828

Abbreviation: CARS: Childhood Autism Rating Scale.

### 3.1 Frequency of Participation

**FIGURE 1:**
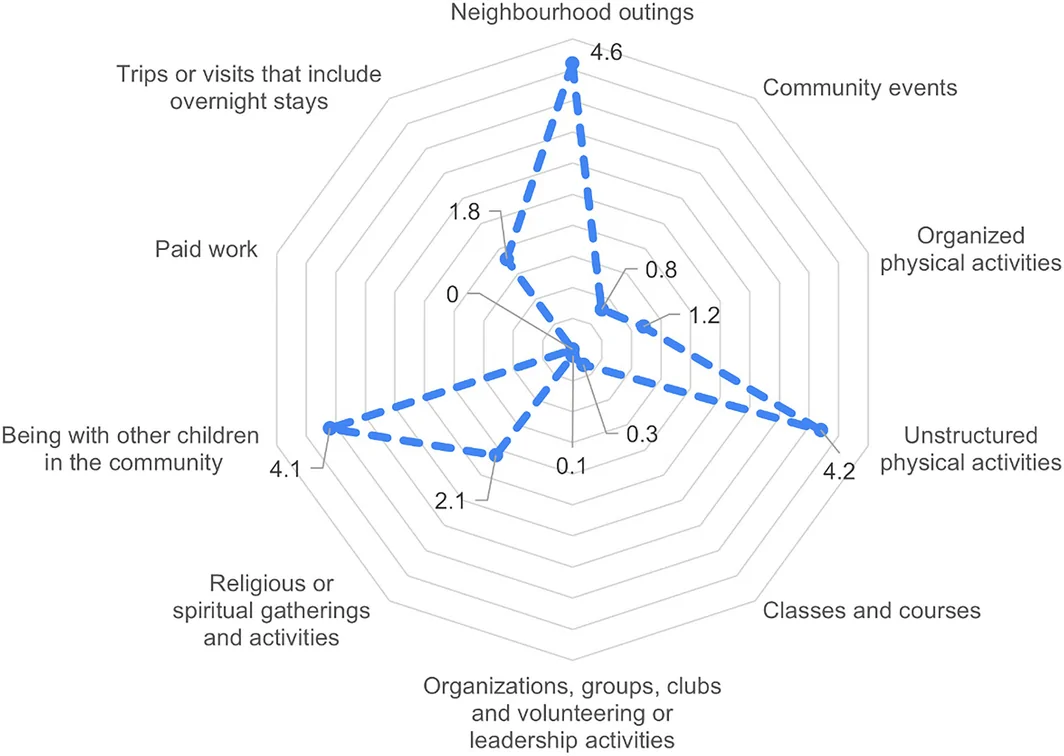
Mean score of frequency of participation at community for each Participation and Environment Measure‐Children and Youth (PEM‐CY) item (ranging from 0 to 7).

The mean frequency of participation in community‐based activities was 3.9 on a scale ranging from 0 to 7 (SD = 0.77). Activities with mean participation frequencies above 4.0 included neighborhood outings (mean = 4.6), unstructured physical activities (mean = 4.2), and spending time with other children in the community (mean = 4.1). In contrast, activities with mean participation frequencies below 1.0 included paid work (mean = 0), participation in organizations, groups, clubs, and volunteering or leadership activities (mean = 0.1), classes and courses (mean = 0.3), and community events (mean = 0.8). Mean participation frequencies for all community activities are presented in Figure 1.

#### 3.1.1 Involvement Across Community‐Based Activities

**FIGURE 2:**
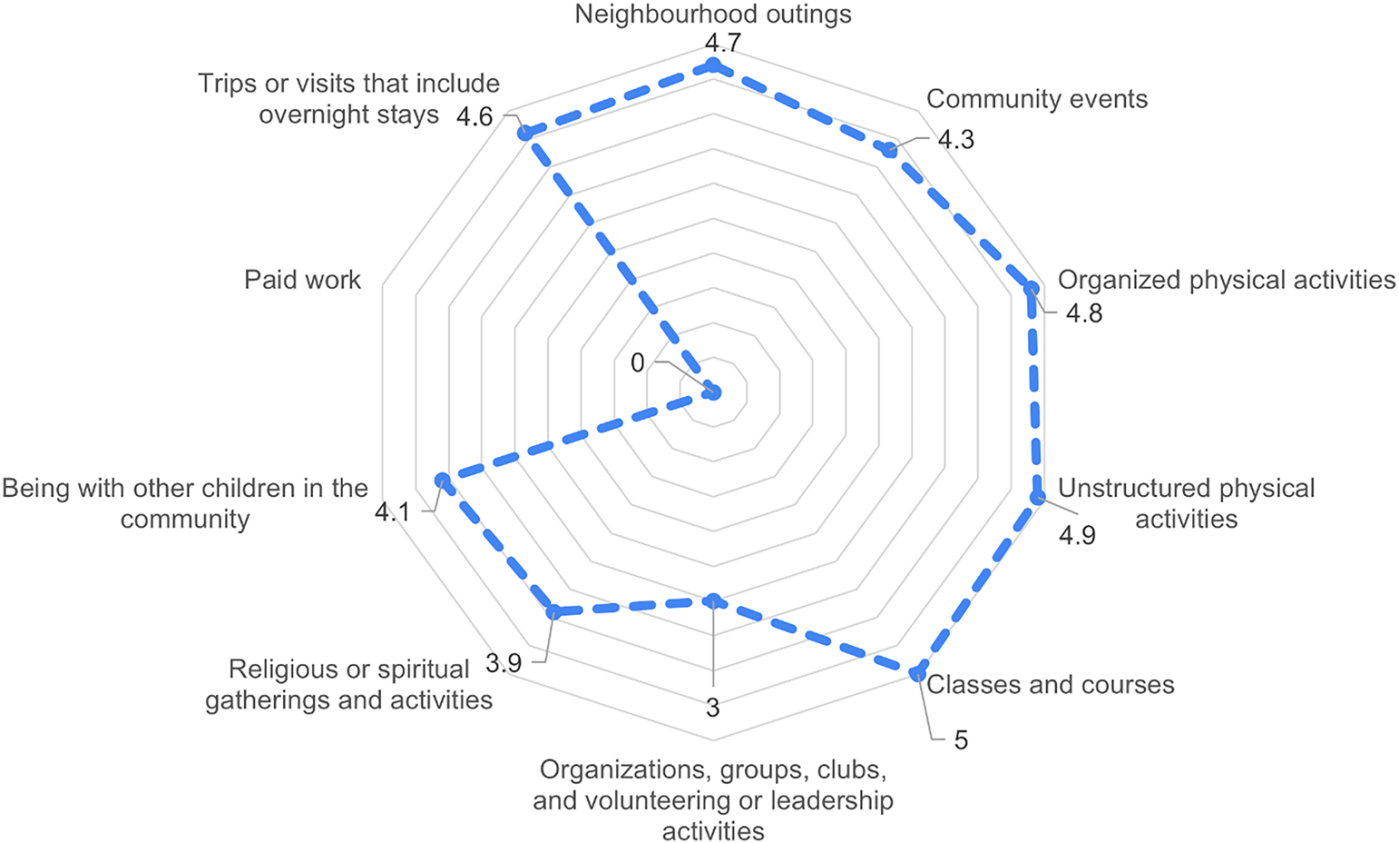
Mean score of involvement in home participation for each Participation and Environment Measure‐Children and Youth (PEM‐CY) item (ranging from 1 to 5).

The mean level of involvement across community‐based activities was 4.5 on a 5‐point scale (SD = 0.63). Activities with mean involvement levels below 4.0 included paid work (mean = 0), participation in organizations, groups, clubs, and volunteering or leadership activities (mean = 3.0), as well as religious or spiritual gatherings and activities (mean = 3.9). Mean involvement levels for all remaining community activities are presented in Figure 2.

#### 3.1.2 Desire for Change

**FIGURE 3:**
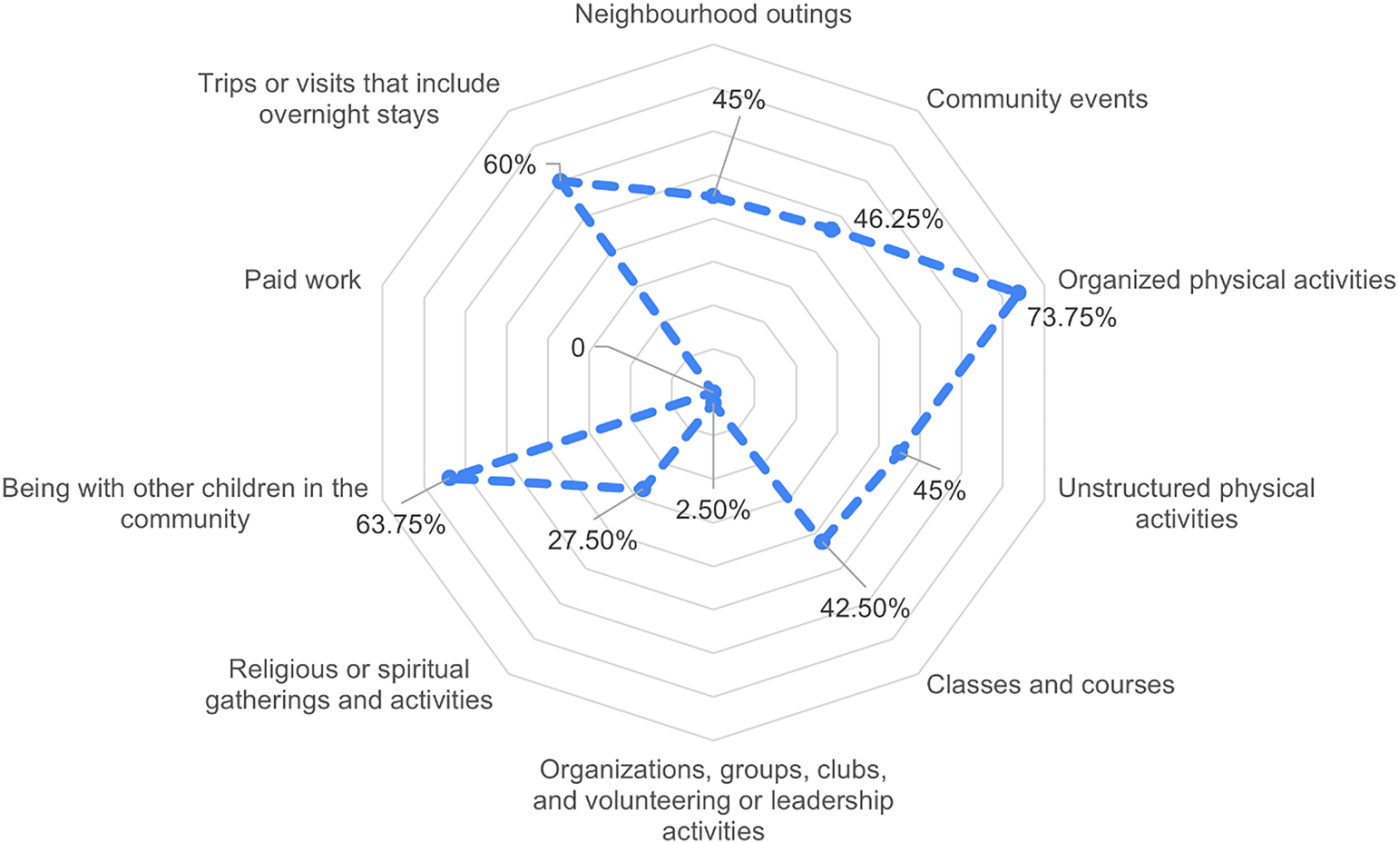
Caregivers' desire for change in community participation for autistic children (total and according to category).

The mean level of caregivers' desire for change across community activities was 46.6%. Activities for which more than half of caregivers expressed a desire for change included organized physical activities (73.8%), spending time with other children in the community (63.8%), and trips or visits involving overnight stays (60%). In contrast, activities with the lowest reported desire for change were paid work (0%), participation in organizations, groups, clubs, and volunteering or leadership activities (2.5%), and religious or spiritual gatherings and activities (2.5%). Mean levels of desire for change for all community activities are presented in Figure 3.

#### 3.1.3 Frequency of Barriers and Environmental Support

**FIGURE 4:**
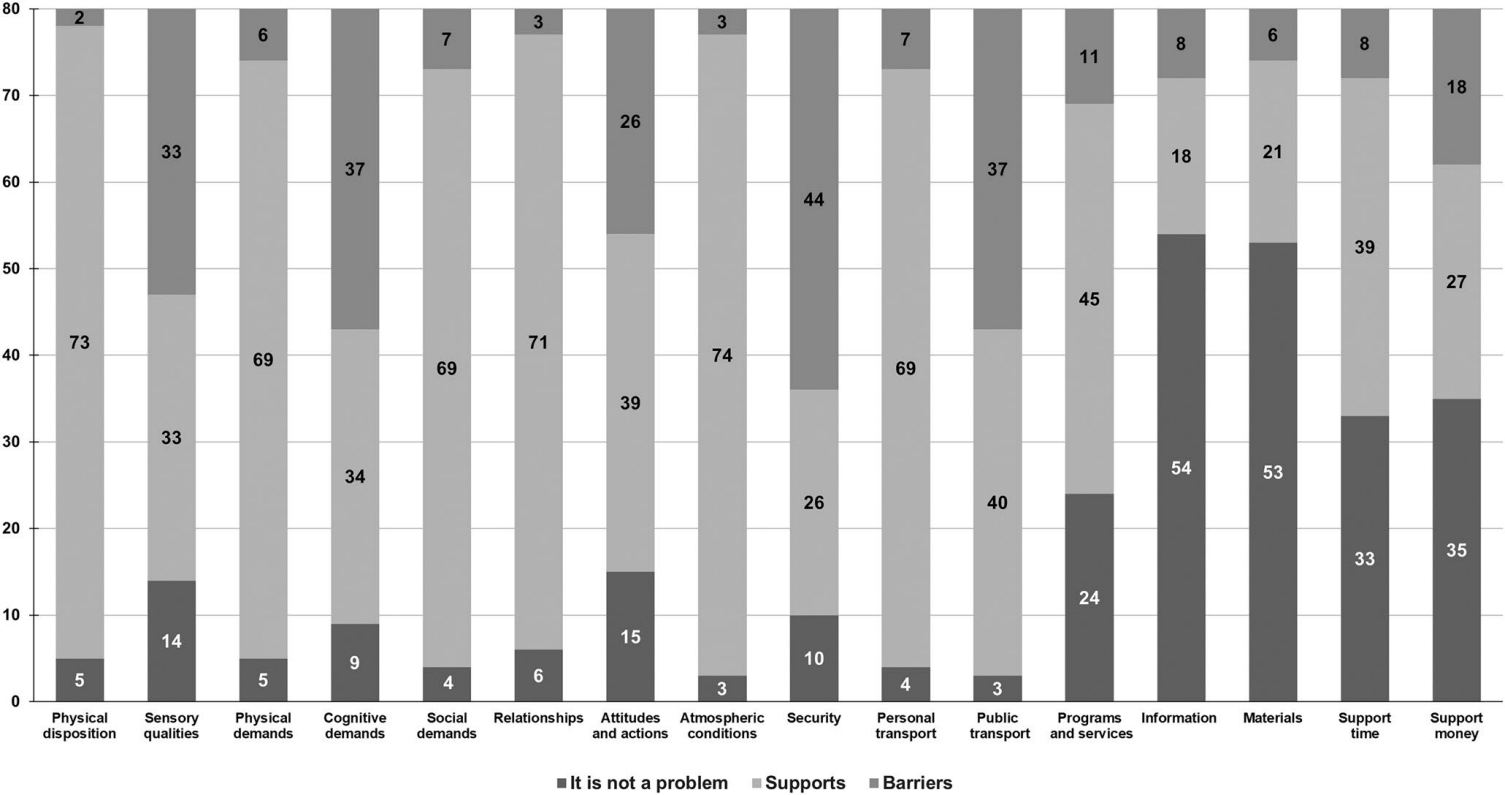
Facilitators, Barriers, and Neutral for each Participation and Environment Measure‐Children and Youth (PEM‐CY) in the community Environment.

In the analysis of environmental factors, caregivers reported an average of 20.4% of items as barriers and 36.0% as support. Environmental supports identified by more than 60 caregivers (75% of the sample) included atmospheric conditions (*n* = 74), physical layout (*n* = 73), interpersonal relationships (*n* = 71), physical demands (*n* = 69), social demands (*n* = 69), and personal transportation (*n* = 69). The most frequently reported environmental barriers were safety (*n* = 44), public transportation (*n* = 37), and cognitive demands (*n* = 37). Sensory qualities were identified as both a support and a barrier by 33 caregivers. These distributions are illustrated in Figure 4.

### 3.2 Path Analysis

The overall model demonstrated an adequate fit to the data (*χ* ^2^ = 16.40, df = 18, *p* = 0.563), indicating good correspondence between the proposed model and the observed data. Model fit indices further supported an excellent fit (RMSEA = 0.00, CFI = 1.00, TLI = 1.00, RNI = 1.00, GFI = 1.00).

Regarding explained variance (*R* ^2^), community participation frequency showed a non‐significant proportion of explained variance (*R* ^2^ = 0.06, *p* = 0.43). Similarly, community participation involvement showed a non‐significant proportion of variance (*R* ^2^ = 0.08, *p* = 0.26) (Table 3).

**TABLE 3:** Direct effects of community environmental factors and family income on participation outcomes.

Outcome	Predictor	*b*	SE	95% CI	*β*	*p*
Frequency	Community barriers (%)	0.01	0.01	[−0.02, 0.04]	0.08	0.446
	Community support (%)	0.02	0.01	[−0.00, 0.03]	0.21	0.065
	Up to R$2.9 thousand	−0.36	0.47	[−1.28, 0.55]	−0.10	0.435
	R$2.9–7.1 thousand	−0.32	0.56	[−1.42, 0.79]	−0.07	0.577
	R$7.1–22 thousand and > R$22 thousand^a^	−0.67	1.28	[−3.18, 1.85]	−0.06	0.604
Involvement	Community barriers (%)	−0.01	0.01	[−0.02, 0.00]	−0.21	0.060
	Community support (%)	0.00	0.00	[−0.00, 0.01]	0.07	0.518
	Up to R$2.9 thousand	0.21	0.17	[−0.13, 0.54]	0.16	0.228
	R$2.9–7.1 thousand	0.33	0.21	[−0.08, 0.74]	0.20	0.110
	R$7.1–22 thousand and *>* R$22 thousand^a^	0.33	0.47	[−0.60, 1.25]	0.08	0.487
Desire for change	Community barriers (%)	−0.15	0.49	[−1.12, 0.82]	−0.03	0.759
	Community support (%)	−0.42	0.31	[−1.04, 0.20]	−0.15	0.181
	Up to R$2.9 thousand	9.09	16.10	[−22.47, 40.65]	0.07	0.572
	R$2.9–7.1 thousand	−6.39	19.49	[−44.59, 31.81]	−0.04	0.743
	R$7.1–22 thousand and > R$22 thousand^a^	3.04	44.31	[−83.80, 89.88]	0.01	0.945

*Note:* Family income categories were based on the socioeconomic classification of the Brazilian Association of Research Companies (ABEP, Associação Brasileira de Empresas de Pesquisa).

Abbreviations: *b*, unstandardized estimates; CI, confidence interval; SE, standard error; *β*, standardized coefficient.

^a^ Because only two participants belonged to the highest income category (>R$22 thousand/month), this category was combined with the adjacent category for the path analysis.

No statistically significant associations were observed between community environmental factors, family income, and participation frequency. Community environmental support showed the largest positive standardized coefficient for participation frequency (*β* = 0.21, *p* = 0.065), whereas community environmental barriers and family income showed smaller effects.

For community participation involvement, community environmental barriers showed the largest negative standardized coefficient (*β* = −0.21, *p* = 0.065), whereas community environmental support and family income were not significantly associated with involvement.

For caregivers' desire for change in community participation, neither environmental factors nor family income showed statistically significant associations. Although a significant covariance was observed between community participation frequency and involvement, no significant paths were identified between the examined predictors and the desire for change.

### 3.3 Family Engagement in the Interpretation of Results

In the qualitative phase, a Family Engagement in the Interpretation of Results approach was adopted, in which research partners were actively involved in interpreting the quantitative findings. This process led to the identification of three main themes related to community participation: environmental barriers, environmental supports, and desire for change.

Regarding environmental barriers, research partners identified financial constraints and parental safety concerns as primary obstacles. Caregivers reported that the high costs associated with therapies consume a substantial portion of family resources, directly limiting opportunities for engagement in community leisure activities, such as traveling. In addition, in response to these environmental barriers, parental emotional and behavioral responses emerged as protective choice adopted to manage perceived safety concerns and contextual challenges. Research partners highlighted concerns about community programs. Caregivers expressed safety concerns, particularly regarding the lack of trained professionals. As Family partner 5 (FP‐5) emphasized: “I don't trust leaving my daughter there. For example, there are free sports programs, but I don't feel safe because I don't feel they are prepared to handle her, only typical children.”

Environmental supports were primarily associated with public policies, access to services, and individual resources such as transportation that facilitate participation. Research partners emphasized that access to personal transportation (e.g., having a car) and public benefits or financial support for autistic individuals are key facilitators that make community participation more feasible.

The theme “desire for change” reflected priorities for increasing participation, particularly, through more frequent opportunities for travel and interaction with peers, as well as greater access to structured activities such as classes and courses. Research partners also identified a mismatch between children's abilities and environmental readiness: “They even have the capacity, but the places aren't prepared to receive them.” Furthermore, the need for greater awareness among parents of typically developing children was emphasized, as limited societal understanding contributes to exclusion and reinforces the use of protective choices, such as avoiding community outings.

A comprehensive synthesis of these themes, subthemes, and representative quotes is presented in Table 4. The five family research partners were identified using pseudonymous codes (FP1–FP5) to ensure confidentiality.

**TABLE 4:** Themes, subthemes, and representative quotes identified through family‐engaged interpretation of quantitative findings on community participation.

Theme	Subtheme	Example quote
	Restrictions in community participation	FP1—“Frequent participation in neighborhood outings (which is complicated for me), and the children don't understand either (because the adults don't explain)”
Participation	Low frequency of participation	FP4—“I have difficulties in the neighborhood (the biggest barrier is being with other people—social demands), I agree with the desire for change—we want them to participate like typical children”
	High involvement in structured activities	FP2—“My son participates in at least two organized physical activities per week; he enjoys them and they are very helpful”
	Attitudinal and social barriers	FP4—“People's attitudes and actions towards him make it difficult; sometimes you stop going out just thinking about it, and they even touch or stress him (son)”
	Service and system‐related barriers	FP5—“I think the lack of accessibility, knowledge among professionals, awareness among children, and inclusion for her to participate like other children, all influence her participation in the community”
	Environmental safety barriers	FP5—“I don't trust leaving my daughter there. For example, there are free sports programs, but I don't feel safe because I don't feel they are prepared to handle her, only typical children”
Environment barriers	Lack of governmental support	FP2—“Financial difficulties due to a lack of government support for programs and services, and difficulties in improving security”
	Transportation barriers	FP2—“I have difficulty traveling to therapy sessions because they are not available in my city”
	Financial barriers	FP1—“Although our household income is sufficient, the costs related to healthcare—such as consultations and specialized therapies that are not available through the public system—limit our ability to engage in community activities. As a result, we often give up opportunities such as traveling, which could otherwise allow my child to interact with other children.”
	Access to transportation	FP1—“Having a car helps”
Environment support	Public policies for autistic individuals (benefits)	FP1—“Having access to discounts because my child is autistic, and having a car, makes participation much easier.”
Personal factors	Parental protective behaviors	FP5—“Sometimes the biggest barriers are us parents! I often avoid going out so I don't have to keep explaining myself and go through difficult situations and feeling insecure. If the community had greater access to information and inclusion, it would make things easier for us parents”
	Increased participation with peers and greater opportunities for travel	FP3—“My desired change: to be with more children and travel more”
Desire for change	Improved access to activities in classes, courses, and organized physical activities	FP4—“Classes and courses are a major area where change is needed. They even have the capacity, but the places aren't prepared to receive them”
	Greater societal awareness	FP3—“It's important to raise awareness among people, as some people simply don't understand”

Abbreviation: FP: family partner.

## 4 Discussion

The present findings are consistent with previous research indicating that autistic children tend to experience distinct participation patterns, particularly in community and socially oriented contexts (Egilson et al. 2017; Askari et al. 2015; Ratcliff et al. 2018). These patterns suggest that participation is not determined solely by child characteristics but rather emerges from dynamic interactions between the child and the environments in which activities occur.

Caregivers reported a low to moderate frequency of community participation, with relatively higher engagement in neighborhood outings and interactions with other children, and substantially lower participation in activities such as overnight travel, classes or courses, and organized physical activities. These findings are consistent with previous research indicating that community environments are often less predictable and less accessible, which may limit participation opportunities for autistic children (Askari et al. 2015; Ratcliff et al. 2018; Budavari et al. 2021). Despite this reduced frequency, involvement during participation was high, in agreement with prior studies showing that when participation occurs, autistic children tend to engage meaningfully (Lamash et al. 2020; Chen et al. 2023; Folta et al. 2022). Together, these findings suggest that restricted participation is more closely related to contextual constraints than to reduced motivation or capacity.

No significant associations were identified between participation outcomes and personal factors, including child age, sex, autism severity (CARS‐BR), or parental education. Although this finding contrasts with studies reporting associations between child characteristics and participation (Egilson et al. 2017; Ratcliff et al. 2018; Salters and Scharoun Benson 2026; Folta et al. 2022), it indicates that the influence of personal factors may be context dependent. For example, Ratcliff et al. (Ratcliff et al. 2018) identified age‐related differences in participation, while Salters and Scharoun Benson (Salters and Scharoun Benson 2026) reported consistent associations between social skills and participation across domains. In the present study, the absence of such associations may be partly explained by methodological and contextual factors, including the use of caregiver‐reported measures within specific ecological contexts and the relative homogeneity and restricted variability of the sample.

These findings are consistent with a biopsychosocial perspective in which participation is understood as the result of interactions between personal and environmental factors (Imms et al. 2017; Anaby et al. 2014). In this context, environmental conditions may influence the extent to which individual characteristics are expressed in participation. Although previous studies have reported negative associations between autism severity and participation (Egilson et al. 2017; Krieger et al. 2024), our findings suggest that, within our sample and specifically in the community context, autism severity was not independently associated with participation outcomes after adjustment for the variables included in the model. This highlights the importance of considering contextual and environmental influences when interpreting participation.

Path analysis did not reveal statistically significant associations between environmental support, barriers, or family income and participation outcomes. This absence of significant paths should not be interpreted as evidence that environmental factors are not influential. Previous literature indicates that participation in autistic children is influenced by multiple interrelated factors that operate concurrently. The scoping review by Askari et al. (Askari et al. 2015) highlights the contribution of environmental accessibility, social context, and individual characteristics to participation. Similarly, Lamash et al. (Lamash et al. 2020) and Ratcliff et al. (Ratcliff et al. 2018) describe participation patterns as variable across contexts and activity types, while Salters and Scharoun Benson (Salters and Scharoun Benson 2026) emphasize the combined influence of social skills, environmental opportunities, and family context.

In this context, the absence of significant direct effects may reflect limitations in representing environmental influences through aggregated indicators and linear relationships. Environmental factors may operate through indirect or conditional pathways that were not captured in the model. In addition, the relatively low frequency of participation observed in this sample may have reduced variability in exposure to community contexts, thereby limiting the detection of associations. These findings support the interpretation of participation as a context‐dependent construct influenced by multiple interacting factors.

Caregiver reports indicated that environmental supports were more frequently perceived than barriers. Physical characteristics, such as atmospheric conditions and physical layout, were commonly identified as supportive, possibly reflecting the absence of physical impairments in many autistic children. Social demands, interpersonal relationships, and personal transportation were also frequently reported as supports. This differs from previous studies that have identified social demands as prominent barriers (Egilson et al. 2017; Krieger et al. 2024), suggesting variability in how families experience social environments.

In contrast, safety concerns, cognitive demands, and sensory demands were the most frequently reported barriers. Safety reflects concerns related to risk and the demands associated with navigating community environments. Cognitive and sensory demands were identified both as barriers and as supports, which is consistent with the ICF perspective that environmental factors may facilitate or restrict participation depending on their interaction with individual characteristics and contextual conditions (Anaby et al. 2014).

The absence of statistically significant associations between participation and environmental factors in the quantitative phase should be interpreted with caution. The low to moderate participation frequency observed may have limited caregivers' exposure to community environments, thereby constraining their ability to identify and report specific environmental influences.

The involvement of family research partners contributed to the interpretation of these findings. Their reports indicated that, although some activities occur with moderate frequency, participation remains challenging in practice. Activities such as travel, classes or courses, and organized physical activities were described as meaningful and desired, but difficult to access due to environmental barriers. When these activities occurred, children demonstrated high levels of involvement, consistent with quantitative findings and previous studies (Egilson et al. 2017; Jeong et al. 2017).

Research partners identified multiple barriers, including systemic and attitudinal factors such as limited inclusion, lack of preparedness, and negative social attitudes, which restricted the use of community spaces and opportunities for interaction (Ghanouni et al. 2019; Simpson and Adams 2024). Safety and trust were also identified as important concerns, influencing parental decisions to limit participation, in agreement with studies linking perceived insecurity and stigma to reduced participation (Krieger et al. 2024; Dunn et al. 2019; Sari et al. 2023).

Financial and logistical constraints were also reported. The costs associated with therapies and health care, particularly those not covered by public services, limited the resources available for community participation. Transportation difficulties and long travel distances further restricted participation opportunities, consistent with previous findings (Mattinson et al. 2019; Rogge and Janssen 2019).

Both quantitative and qualitative findings identified personal transportation as an important facilitator of participation (Wang et al. 2024). Access to a private vehicle was associated with greater autonomy and flexibility in planning activities. In addition, financial support and access to services were identified as facilitators, particularly, when combined with structured opportunities for participation. These findings are consistent with studies indicating that inclusive policies and environmental adaptations support participation (Edwards et al. 2024).

Research partners expressed a desire for increased participation in activities involving social interaction, organized programs, and community engagement. Greater awareness among parents of typically developing children and broader social inclusion were identified as important conditions for improving participation. These findings are consistent with previous studies emphasizing the role of perceived accessibility and social support (Xu et al. 2025; Sequeira et al. 2023; Wallace‐Watkin et al. 2023).

The integration of caregiver‐reported data and research partners' perspectives suggests that low participation frequency may reflect a response to environments perceived as unsafe, inaccessible, or insufficiently prepared. In this context, restricted participation appears to be more closely related to environmental conditions than to a lack of motivation or capacity, consistent with the high levels of involvement and the reported desire for change.

## 5 Limitations

Several limitations should be acknowledged. The convenience sample, composed primarily of families with access to specialized services, may limit generalizability. Cognitive and functional profiles were not controlled for and may moderate the relationship between environmental demands and participation. Additionally, reliance on caregiver reports may introduce perception bias, particularly, in community contexts where caregivers may have limited direct involvement. A methodological limitation of the present study is the relatively small sample size (*N* = 80) utilized for the path analysis. In structural equation modeling, small samples can constrain statistical power, limiting the ability to detect discrepancies between the hypothesized model and the observed data. Consequently, the optimal model fit achieved in our analysis must be interpreted with caution, as it may reflect a lack of statistical power to detect subtle misfits rather than unequivocal evidence of an optimal model specification. Future research employing larger sample sizes is warranted to cross‐validate these findings and rigorously test the robustness of the identified structural pathways.

## 6 Conclusion

Participation in community settings emerged as an ecological and context‐dependent construct shaped by interactions between children, families, and environments. The findings suggest that participation restrictions among autistic children may be less attributable to individual characteristics and more closely related to community environments that remain insufficiently inclusive and responsive. By translating broad environmental barriers into specific social and structural factors, this study underscores the need for interventions that focus not only on children and families, but also on promoting more inclusive and accessible community environments to support meaningful participation.

## Author Contributions

**Léia Cordeiro de Oliveira:** methodology, research, formal analysis and writing – preparation of the original draft. **Cid André Fidelis de Paula Gomes:** writing – review and editing. **Gustavo Pietracatelli Janizello:** formal quantitative research and analysis. **Wallace Pereira Silva:** quantitative analysis. **Alice Soper:** quantitative methodology and review. **Monika Novak‐Pavlic:** quantitative methodology. **Olaf Kraus de Camargo:** review. **Soraia Micaela Silva:** writing – review and editing.

## Funding

This work was partially supported by *Coordenação de Aperfeiçoamento de Pessoal de Nível Superior* (CAPES), finance code 001.

## Ethics Statement

The proposal for this study was analyzed and approved by the Research Ethics Committee of Universidade Nove de Julho (CoEP‐UNINOVE), São Paulo, Brazil, under opinion: CAAE: 70424923.1.0000.5511.

## Consent

Written informed consent was obtained from all parents/caregivers who participated in the study.

## Conflicts of Interest

The authors declare no conflicts of interest.

## Data Availability

The data that support the findings of this study are available on request from the corresponding author. The data are not publicly available due to privacy or ethical restrictions.

## References

[aur70316-bib-0001] American Psychiatric Association , ed. 2013. Diagnostic and Statistical Manual of Mental Disorders. 5th ed. American Psychiatric Association.

[aur70316-bib-0002] Anaby, D. , M. Law , W. Coster , et al. 2014. “The Mediating Role of the Environment in Explaining Participation of Children and Youth With and Without Disabilities Across Home, School, and Community.” Archives of Physical Medicine and Rehabilitation 95, no. 5: 908–917. 10.1016/j.apmr.2014.01.005. 24468018

[aur70316-bib-0003] Askari, S. , D. Anaby , M. Bergthorson , A. Majnemer , M. Elsabbagh , and L. Zwaigenbaum . 2015. “Participation of Children and Youth With Autism Spectrum Disorder: A Scoping Review.” Review Journal of Autism and Developmental Disorders 2, no. 1: 103–114. 10.1007/s40489-014-0040-7.

[aur70316-bib-0004] Bedell, G. , W. Coster , M. Law , et al. 2013. “Community Participation, Supports, and Barriers of School‐Age Children With and Without Disabilities.” Archives of Physical Medicine and Rehabilitation 94, no. 2: 315–323. 10.1016/j.apmr.2012.09.024. 23044364

[aur70316-bib-0005] Braun, V. , and V. Clarke . 2019. “Reflecting on Reflexive Thematic Analysis.” Qualitative Research in Sport, Exercise and Health 11, no. 4: 589–597. 10.1080/2159676X.2019.1628806.

[aur70316-bib-0006] Budavari, A. C. , E. T. Pas , G. F. Azad , and H. E. Volk . 2021. “Sitting on the Sidelines: Disparities in Social, Recreational, and Community Participation Among Adolescents With Autism Spectrum Disorder.” Journal of Autism and Developmental Disorders 52: 3399–3412. 10.1007/s10803-021-05216-0. PMC8801542 34331628

[aur70316-bib-0007] Chen, Y. J. , E. Duku , A. Zaidman‐Zait , et al. 2023. “Variable Patterns of Daily Activity Participation Across Settings in Autistic Youth: A Latent Profile Transition Analysis.” Autism 27, no. 8: 13623613231154729. 10.1177/13623613231154729. PMC10576904 36855223

[aur70316-bib-0008] Coster, W. , G. Bedell , M. Law , et al. 2011. “Psychometric Evaluation of the Participation and Environment Measure for Children and Youth.” Developmental Medicine and Child Neurology 53, no. 11: 1030–1037. 10.1111/j.1469-8749.2011.04094.x. 22014322

[aur70316-bib-0009] Coury, D. L. , D. S. Murray , A. Fedele , T. Hess , A. Kelly , and K. A. Kuhlthau . 2020. “The Autism Treatment Network: Bringing Best Practices to All Children With Autism.” Pediatrics 145, no. Suppl 1: S13–S19. 10.1542/2019-1895D. 32238527

[aur70316-bib-0010] Cross, A. , A. K. Soper , D. Thomson , et al. 2024. “Development, Implementation, and Scalability of the Family Engagement in Research Course: A Novel Online Course for Family Partners and Researchers in Neurodevelopmental Disability and Child Health.” Research Involvement and Engagement 10, no. 1: 80. 10.1186/s40900-024-00561-x. PMC11302057 39103968

[aur70316-bib-0011] de Oliveira, L. C. , G. P. Janizello , I. S. Santos , et al. 2025. “Systematic Review of Outcome Measures Used in Support Programs Designed to Enhance the Functioning for Autistic Children and Adolescents and ICF Content Mapping.” Disability and Rehabilitation 47, no. 17: 4316–4340. 10.1080/09638288.2025.2450050. 39797616

[aur70316-bib-0012] den Houting, J. , J. Higgins , K. Isaacs , J. Mahony , and E. Pellicano . 2022. “From Ivory Tower to Inclusion: Stakeholders' Experiences of Community Engagement in Australian Autism Research.” Frontiers in Psychology 13: 876990. 10.3389/fpsyg.2022.876990. PMC9454607 36092113

[aur70316-bib-0013] Dunn, A. , M. Zarrinkoub , and J. Blacher . 2019. “Social Support and Stress Among Mothers of Children With Autism Spectrum Disorder: A Systematic Review.” Journal of Intellectual & Developmental Disability 44, no. 4: 462–475. 10.3109/13668250.2017.1415170.

[aur70316-bib-0014] Edwards, C. , T. Tutton , and V. Gibbs . 2024. “Organized Physical Activity Participation Among Autistic Australians: Barriers, Enablers and Implications for Inclusion.” Neurodiversity 2: 2. 10.1177/27546330241240648.

[aur70316-bib-0015] Egilson, S. T. , G. Jakobsdóttir , and L. B. Ólafsdóttir . 2017. “Community Participation and Environment of Children With and Without Autism Spectrum Disorder: Parent Perspectives.” Scandinavian Journal of Educational Research 24, no. 3: 187–196. 10.1080/11038128.2016.1198419. 27329683

[aur70316-bib-0016] Egilson, S. T. , G. Jakobsdóttir , and L. B. Ólafsdóttir . 2018. “Parent Perspectives on Home Participation of High‐Functioning Children With Autism Spectrum Disorder Compared With a Matched Group of Children Without Autism Spectrum Disorder.” Autism 22: 560–570. 10.1177/1362361316685555. 28429605

[aur70316-bib-0017] Eidson, T. , A. Hess , T. Hess , and A. Kelly . 2020. “Family Engagement in the Autism Treatment and Learning Health Networks.” Pediatrics 145, no. Suppl 1: S30–S34. 10.1542/peds.2019-1895F. 32238529

[aur70316-bib-0018] Fernandes, A. C. , D. O. Souto , R. R. de Sousa Junior , et al. 2023. “Sports Stars Brazil in Children With Autism Spectrum Disorder: A Feasibility Randomized Controlled Trial Protocol.” PLoS One 18, no. 11: e0291488. 10.1371/journal.pone.0291488. PMC10631688 37939077

[aur70316-bib-0019] Folta, S. C. , L. G. Bandini , A. Must , J. Pelletier , K. Ryan , and C. Curtin . 2022. “Exploring Leisure Time Use and Impact on Well‐Being Among Transition‐Age Autistic Youth.” Research in Autism Spectrum Disorder 96: 101996. 10.1016/j.rasd.2022.101996.

[aur70316-bib-0020] Frisch, N. , P. Atherton , M. M. Doyle‐Waters , et al. 2020. “Patient‐Oriented Research Competencies in Health (PORCH) for Researchers, Patients, Healthcare Providers, and Decision‐Makers: Results of a Scoping Review.” Research Involvement and Engagement 6: 4. 10.1186/s40900-020-0180-0. PMC7011284 32055415

[aur70316-bib-0021] Galvão, E. R. V. P. , A. P. M. Cazeiro , A. C. De Campos , and E. Longo . 2018. “Medida da Participação e do Ambiente ‐ Crianças e Jovens (PEM‐CY): adaptação transcultural para o uso no Brasil.” Revista de Terapia Ocupacional da Universidade de São Paulo 29, no. 3: 237–245. 10.11606/issn.2238-6149.v29i3p237-245.

[aur70316-bib-0022] Ghanouni, P. , T. Jarus , J. G. Zwicker , J. Lucyshyn , S. Chauhan , and C. Moir . 2019. “Perceived Barriers and Existing Challenges in Participation of Children With Autism Spectrum Disorders: “He Did Not Understand and No One Else Seemed to Understand Him”.” Journal of Autism and Developmental Disorders 49, no. 8: 3136–3145. 10.1007/s10803-019-04036-7. 31049788

[aur70316-bib-0023] Guichard, S. , and C. Grande . 2019. “The Role of Environment in Explaining Frequency of Participation of Pre‐School Children in Home and Community Activities.” International Journal of Developmental Disabilities 65: 108–115. 10.1080/20473869.2017.1378160. PMC8115556 34141330

[aur70316-bib-0024] Im, D. , J. Pyo , H. Lee , H. Jung , and M. Ock . 2023. “Qualitative Research in Healthcare: Data Analysis.” Journal of Preventive Medicine and Public Health 56, no. 2: 100–110. 10.3961/jpmph.22.471. PMC10111102 37055353

[aur70316-bib-0025] Imms, C. , M. Granlund , P. H. Wilson , B. Steenbergen , P. L. Rosenbaum , and A. M. Gordon . 2017. “Participation, Both a Means and an End: A Conceptual Analysis of Processes and Outcomes in Childhood Disability.” Developmental Medicine and Child Neurology 59: 16–25. 10.1111/dmcn.13237. 27640996

[aur70316-bib-0026] Jeong, Y. , M. Law , P. Stratford , C. DeMatteo , and C. Missiuna . 2017. “Measuring Children's Participation and Environmental Factors at Home, School and Community: Construct Validation of the Korean PEM‐CY.” Physical & Occupational Therapy in Pediatrics 37: 541–554. 10.1080/01942638.2017.1280870. 28266879

[aur70316-bib-0027] Jesus, C. , I. C. Rodrigues Regalado , I. Menezes , et al. 2025. “Better Together: Participatory Action Research for Co‐Constructing an Intervention to Enhance Leisure Activities in Non‐Ambulatory Adolescents With Cerebral Palsy.” Research Involvement and Engagement 11, no. 1: 69. 10.1186/s40900-025-00684-5. PMC12180169 40542452

[aur70316-bib-0028] Kaelin, V. C. , S. Saluja , D. L. Bosak , D. Anaby , M. Werler , and M. A. Khetani . 2024. “Caregiver Strategies Supporting Community Participation Among Children and Youth With or at Risk for Disabilities: A Mixed‐Methods Study.” Frontiers in Pediatrics 12: 1345755. 10.3389/fped.2024.1345755. PMC10902462 38425659

[aur70316-bib-0029] Krieger, B. , A. Moser , T. Morgenthaler , A. J. H. M. Beurskens , and B. Piškur . 2024. “Parents' Perceptions: Environments and the Contextual Strategies of Parents to Support the Participation of Children and Adolescents With Autism Spectrum Disorder‐A Descriptive Population‐Based Study From Switzerland.” Journal of Autism and Developmental Disorders 54, no. 3: 871–893. 10.1007/s10803-022-05826-2. PMC9765345 36538129

[aur70316-bib-0030] Kwon, S. C. , S. D. Tandon , N. Islam , L. Riley , and C. Trinh‐Shevrin . 2018. “Applying a Community‐Based Participatory Research Framework to Patient and Family Engagement in the Development of Patient‐Centered Outcomes Research and Practice.” Translational Behavioral Medicine 8, no. 5: 683–691. 10.1093/tbm/ibx026. PMC6128966 30202926

[aur70316-bib-0031] Lamash, L. , G. Bedell , and N. Josman . 2020. “Participation Patterns of Adolescents With Autism Spectrum Disorder Compared to Their Peers: Parents' Perspectives.” British Journal of Occupational Therapy 83, no. 2: 78–87. 10.1177/0308022619853518.

[aur70316-bib-0032] Malta, M. , L. O. Cardoso , F. I. Bastos , M. M. F. Magnanini , and C. M. F. P. Silva . 2010. “Iniciativa STROBE: subsídios para a comunicação de estudos observacionais.” Revista de Saúde Pública 44, no. 3: 559–565. 10.1590/S0034-89102010000300021. 20549022

[aur70316-bib-0033] Mattinson, S. , M. Falkmer , M. H. Black , and S. Girdler . 2019. “Participation Profiles and the Barriers and Facilitators That Impact on Participation of Children With Autism Spectrum Disorders Living in Regional and Remote Western Australia.” Scandinavian Journal of Child and Adolescent Psychiatry and Psychology 6: 170–182. 10.21307/sjcapp-2018-018. PMC7852351 33907690

[aur70316-bib-0034] Miot, H. A. 2011. “Tamanho da amostra em estudos clínicos e experimentais.” Jornal Vascular Brasileiro 10, no. 4: 275–278. 10.1590/S1677-54492011000400001.

[aur70316-bib-0035] Novak‐Pavlic, M. , P. Rosenbaum , and B. Di Rezze . 2023. “Changing Directions and Expanding Horizons: Moving Towards More Inclusive Healthcare for Parents of Children With Developmental Disabilities.” International Journal of Environmental Research and Public Health 20: 6983. 10.3390/ijerph20216983. PMC10649410 37947541

[aur70316-bib-0036] Pereira, A. , R. S. Riesgo , and M. B. Wagner . 2008. “Childhood Autism: Translation and Validation of the Childhood Autism Rating Scale for Use in Brazil.” Jornal de Pediatria 84, no. 6: 487–494. 10.2223/JPED.1828. 18923798

[aur70316-bib-0037] Pyo, J. , W. Lee , E. Y. Choi , S. G. Jang , and M. Ock . 2023. “Qualitative Research in Healthcare: Necessity and Characteristics.” Journal of Preventive Medicine and Public Health 56, no. 1: 12–20. 10.3961/jpmph.22.451. PMC9925284 36746418

[aur70316-bib-0038] Ratcliff, K. , I. Hong , and C. Hilton . 2018. “Leisure Participation Patterns for School‐Age Youth With Autism Spectrum Disorders: Findings From the 2016 National Survey of Children's Health.” Journal of Autism and Developmental Disorders 48, no. 11: 3783–3793. 10.1007/s10803-018-3643-5. PMC6482826 29909498

[aur70316-bib-0039] Retamal‐Walter, F. , M. Waite , and N. Scarinci . 2023. “Families' and Professionals' Perspectives of Building and Maintaining Engagement in Telepractice Early Intervention for Young Children With Communication Disability.” Disability and Rehabilitation 45, no. 7: 1165–1177. 10.1080/09638288.2022.2055161. 35348401

[aur70316-bib-0040] Rogge, N. , and J. Janssen . 2019. “The Economic Costs of Autism Spectrum Disorder: A Literature Review.” Journal of Autism and Developmental Disorders 49, no. 7: 2873–2900. 10.1007/s10803-019-04014-z. 30976961

[aur70316-bib-0041] Rosseel, Y. 2012. “Lavaan: An R Package for Structural Equation Modeling.” Journal of Statistical Software 48, no. 2: 1–36. 10.18637/jss.v048.i02.

[aur70316-bib-0042] Salters, D. , and S. Scharoun Benson . 2026. “Factors Associated With Participation in Different Activities Among Children With ASD: Caregiver Perspectives of Participation.” Journal of Occupational Therapy, Schools & Early Intervention 19, no. 1: 60–77. 10.1080/19411243.2025.2463362.

[aur70316-bib-0043] Sari, G. C. , Z. Karasu , and H. Y. Çiftci . 2023. “Perceived Stigma in Community‐Based Leisure Activity Participation of Children With Autism: Perspective From Turkish Parents.” Journal of Pediatric Nursing 68: e54–e60. 10.1016/j.pedn.2022.10.007.

[aur70316-bib-0044] Sequeira, E. , S. O'Brien , and A. Shimmick . 2023. “Perceived Neighborhood Accessibility and Child Participation in Community‐Based Activities: Perspectives of Mothers of Children With Autism Spectrum Disorder.” Journal of Autism and Developmental Disorders 53, no. 1: 329–339. 10.1007/s10803-021-05386-x.

[aur70316-bib-0045] Simpson, K. , and D. Adams . 2024. “Parent‐Reported Environmental Factors and Strategies to Support Home and Community Participation in Children on the Autism Spectrum.” Disability and Rehabilitation 46, no. 17: 3970–3979. 10.1080/09638288.2023.2261843. 37772748

[aur70316-bib-0046] Singh, O. P. 2021. “Comprehensive Mental Health Action Plan 2013‐2030: We Must Rise to the Challenge.” Indian Journal of Psychiatry 63, no. 5: 415–417. 10.4103/indianjpsychiatry.indianjpsychiatry_811_21. PMC8522612 34789927

[aur70316-bib-0047] Smits, D. W. , K. van Meeteren , M. Klem , M. Alsem , and M. Ketelaar . 2020. “Designing a Tool to Support Patient and Public Involvement in Research Projects: The Involvement Matrix.” Research Involvement and Engagement 6, no. 6: 30. 10.1186/s40900-020-00188-4. PMC7296703 32550002

[aur70316-bib-0048] Taheri, A. , A. Perry , and P. Minnes . 2016. “Examining the Social Participation of Children and Adolescents With Intellectual Disabilities and Autism Spectrum Disorder in Relation to Peers.” Journal of Intellectual Disability Research 60, no. 5: 435–443. 10.1111/jir.12289. 27120987

[aur70316-bib-0049] Verdecias‐Pellum, N. , C. Silverman , M. Yudell , and A. Carroll‐Scott . 2025. “Inequities in Community‐Engaged Autism Research: Community Member Perspectives.” Progress in Community Health Partnerships 19, no. 1: 25–34. 10.1353/cpr.2025.a956594. 40223625

[aur70316-bib-0050] Wallace‐Watkin, C. , J. Sigafoos , L. Woods , and H. Waddington . 2023. “Parent Reported Barriers and Facilitators to Support Services for Autistic Children in Aotearoa New Zealand.” Autism 27, no. 8: 13623613231168240. 10.1177/13623613231168240. PMC10576898 37129303

[aur70316-bib-0051] Wang, Z. , A. Golos , J. A. Weiss , and D. Anaby . 2024. “Participation of Children With Autism During COVID‐19: The Role of Maternal Participation.” OTJR 44, no. 1: 13–24. 10.1177/15394492231164939. PMC10125891 37089012

[aur70316-bib-0052] Whybrow, S. , B. Milbourn , B. Afsharnejad , C. Kasari , S. Bölte , and S. Girdler . 2025. “Where Are the Children's Voices? A Scoping Review of Autistic Children's Participation in Primary School Recess.” Review Journal of Autism and Developmental Disorders. 10.1007/s40489-025-00518-w.

[aur70316-bib-0053] Williams, U. , M. Law , S. Hanna , and J. W. Gorter . 2019. “Using the Young Children's Participation and Environment Measure (YC‐PEM) to Describe Young Children's Participation and Relationship to Disability and Complexity.” Journal of Developmental and Physical Disabilities 31: 135–148. 10.1007/s10882-018-9637-6.

[aur70316-bib-0054] Xu, J. , G. Graziosi , F. F. Rodrigues , R. Tian , R. Birnbaum , and N. Neil . 2025. “Economic Impacts of Caring for Autistic Children in Ontario, Canada: Report From a Pilot Study.” Frontiers in Public Health 13: 1659801. 10.3389/fpubh.2025.1659801. PMC12484171 41041377

[aur70316-bib-0055] Zeidan, J. , E. Fombonne , J. Scorah , et al. 2022. “Global Prevalence of Autism: A Systematic Review Update.” Autism Research 15, no. 5: 778–790. 10.1002/aur.2696. PMC9310578 35238171

